# Light People: Professor Lin Li

**DOI:** 10.1038/s41377-021-00566-x

**Published:** 2021-07-19

**Authors:** Hui Wang, Heng Gu

**Affiliations:** 1 Department of International Cooperation, Changchun Institute of Optics, Fine Mechanics and Physics, Chinese Academy of Sciences, 3888 Dong Nan Hu Road, 130033 Changchun, China; 2 School of Mechanical Engineering, Jiangsu University, Jiang Su, China

**Keywords:** Lasers, LEDs and light sources, Physics

## Abstract

How to deal with climate change, how to mitigate or even reverse it, maybe the hottest scientific topic of the 21st century. Do you know that a Chinese scientist and his team contributed to climate change control by reducing PM2.5 in diesel car exhaust by 35–40%? That scientist is Prof. Lin Li, a Fellow of the Royal Academy of Engineering and founder of the Laser Processing Research Centre at The University of Manchester, United Kingdom. Of course, this is only one example of Professor Li’s scientific achievements. As a pioneer of microsphere super-resolution lens, his team, in collaboration with Singapore colleagues, broke the optical diffraction limit in optical microscopic imaging, making real-time observation of biological viruses without interference possible. He also used lasers to synthesize new nanomaterials which kill drug-resistant bacteria while remaining harmless to healthy human cells, which led to the development and breakthrough of related research fields. We are much honored to have Professor Lin Li for an exclusive interview in which he recalls his years of scientific research experience and talks about the future development trend of laser material processing.

## Abstract (web summary)

A pioneer in laser material processing and super-resolution microsphere lens imaging, Prof. Lin Li’s achievements not only lead to advancing the scientific fields but also applications in our daily life.

## Graphical abstract

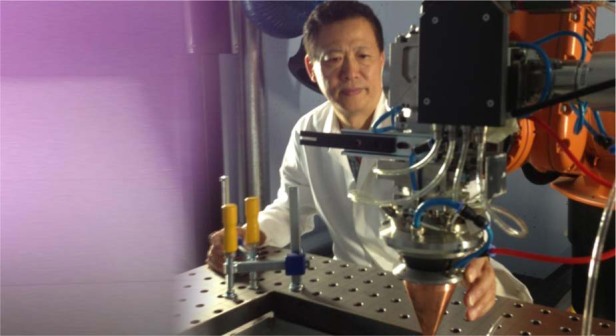



**Biography**: Professor Lin Li is Director of Laser Processing Research Centre at The University of Manchester, UK. He served as President of Laser Institute of America (2016), President of International Academy of Photonics and Laser Engineering (2013–2015), and President of Association of Industrial Laser Users (AILU–2017–2019).

He is an elected Fellow of Royal Academy of Engineering (FREng), Fellow of Laser Institute of America (LIA), Fellow of International Academy of Production Engineering (CIRP), Fellow of International Academy of Photonics and Laser Engineering (IAPLE), and Fellow of Institute of Engineering and Technology (IET). He is an author and co-author of over 400 publications in peer-reviewed journals related to laser-based manufacturing and an inventor or co-inventor of more than 60 patents. He received Sir Frank Whittle Medal from the Royal Academy of Engineering in 2013 for innovative manufacturing that has generated significant economic impacts. In 2014 he received Wolfson Research Merit Award from the Royal Society for his research on laser nano-fabrication and nano-imaging and received the Researcher of the Year medal from The University of Manchester in 2014. He received the Arthur Schawlow award from Laser Institute of America in 2019 for distinguished achievements in laser-based manufacturing, and the Donald Julius Groen Prize, and the Arthur Charles Main Award from the Institution of Mechanical Engineers for leadership in additive manufacturing.

He obtained a BSc degree in Control Engineering from Dalian University of Technology (China) in 1982 and a Ph.D. degree in Laser Engineering from Imperial College London in 1989. He worked at The University of Liverpool (UK) between 1988 and 1994 as a Research Associate and Research Fellow in laser material processing and became a Lecturer in Manufacturing at The University of Manchester Institute of Science and Technology (UMIST), UK, and was promoted to a Full Professor in Laser Engineering in 2000. He served as the Deputy Head of School of Mechanical, Aerospace and Civil Engineering at The University of Manchester during 2009–2013 and served as the Associate Dean of Faculty of Science and Engineering at The University of Manchester during 2015–2020.

**1. You are a pioneer in laser material processing who has been active in the field of laser drilling, laser welding, laser cutting, laser surface treatment, laser additive manufacturing, laser cleaning, and laser micro-nano manufacturing. Your work has been widely applied in manufacturing, medical, biological, new energy, and many other fields. What do you think are the key challenges facing laser material processing today? And what is its future development trend?**

Lin Li: I started laser processing research in 1985 at Imperial College London in the UK with Professor Bill Steen who is widely recognized as the father of laser material processing. From him, I learned that no matter what you do, curiosity and society’s needs are always the key drivers for research. At that time in the 1980s, lasers were considered as a solution looking for problems. Over the past 40 years, significant advances have been made including ultrafast (picosecond and femtosecond pulsed) lasers and the use of these lasers for micro and nanofabrication. The availability of high-powered fiber lasers has revolutionized laser cutting and welding processes and systems with much higher energy efficiency, processing precision, and flexibility than the previously used CO_2_ and Nd:YAG lasers. Additive manufacturing of metallic components using high-power lasers has evolved from a laboratory rapid prototyping tool to a widely recognized and applied rapid manufacturing tool, saving energy, cost, materials, and time. Today, we are still facing many scientific and technological challenges in laser processing. One challenge is the application and integration of artificial intelligence and in-process sensors in laser processing systems and research to allow better process control and innovation. Another challenge is to overcome the micro/nano and macro processing barrier to enable micro/nano processing precision to be realized in macro processes and the efficiency of macro-processing to be realized in micro/nano processes. I gave a keynote presentation at the 31st ICALEO conference held in the USA in 2012 on “The Interface and Crossover between Laser Micro/Nano Fabrication and Macro Processing”. This challenge is still facing us today. The third challenge is to further improve the energy efficiency of laser processing to aim at net zero-emission in laser processes and to enable zero energy waste in laser processing through reflection, and thermal conduction to the bulk energy losses.

**Figure:**
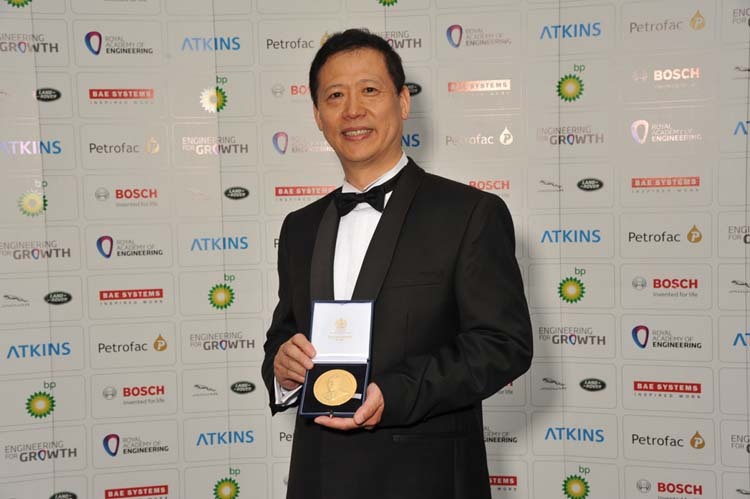
Professor Lin Li received Sir Frank Whittle Medal from the Royal Academy of Engineering in 2013.

**2. Multi-material laser additive manufacturing is a current research hotspot. Recently, your team has developed a laser powder bed fusion (LPBF) additive manufacturing system which utilizes ultrasonic vibration to achieve precise powder delivery during the deposition of different powder materials. Multi-material and functionally graded components made of metal, ceramic, glass, and polymer were successfully fabricated, providing a new solution to achieving multi-material laser additive manufacturing. As a pioneer of this technology, what do you think are the main applications for this technology? What kind of challenges might it face?**

Lin Li: In our lives, hardly any products and tools we use are made of a single material. This is because different materials have different properties and play different roles. In conventional manufacturing, different parts of materials made of different materials are manufactured separately and then they are assembled together using various joining techniques. This requires different machines and operators with multiple steps, thus would consume more energy. Although additive manufacturing or 3D printing has been around for nearly 40 years, today it is still dominated by the use of a single type of materials in each component. In laser-based additive manufacturing, the challenge in combining different types of materials in a single component is that the laser processing will need to tailor for different material properties including melting point, thermal expansion coefficient, thermal conductivity, laser light absorption, and material compatibility to each other. For example, metal and ceramics are completely different types of materials. Joining them together is very challenging. The functional grading approach is used to vary gradually from one material to another in additive manufacturing. This requires multiple material 3D printing technologies. Potential applications of multiple material additive manufacturing include next-generation medical implants with combined high strength, corrosion resistance, light-weight, biocompatibility, controlled biodegradability, and self-monitoring. Another application would be in an aero-engine where high temperature and light-weight are required. A third area would be in impact-resistant personal protection products where lightweight and high-impact resistance are required. Multiple material additive manufacturing is a new field of study. Many material science issues also need to be understood including the control of intermetallic phases.

**Figure:**
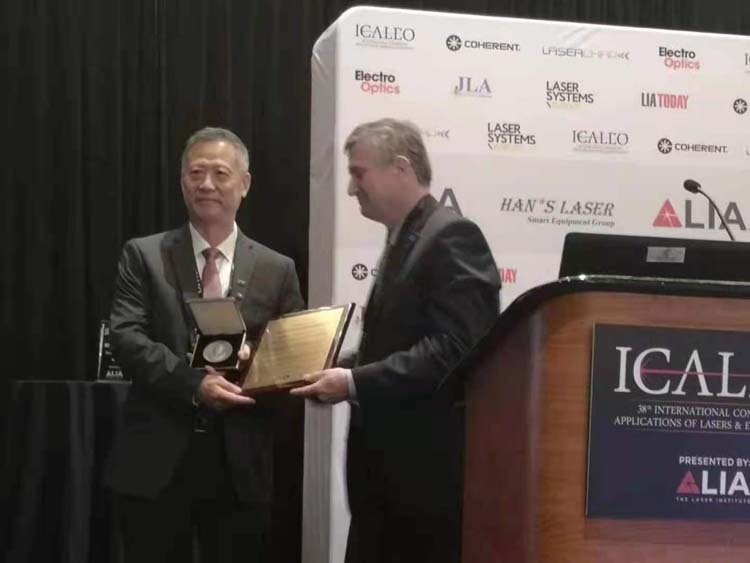
Professor Lin Li received the Arthur Schawlow award from the Laser Institute of America in 2019.

**3. We all know that graphene was first separated at The University of Manchester. In recent years, your team has published many academic papers on laser direct writing of graphene. You have also applied for a patent using this new method of producing graphene. What are the advantages of using lasers to prepare graphene? Did you encounter any difficulties when carrying out this research?**

Lin Li: The common method for producing high-quality graphene is by chemical vapor deposition. It requires a metallic catalyst substrate such as Cu or Ni and carbon-containing gases such as methane to react at high temperatures of over 1000 °C. Then for practical applications, graphene formed in such a way needs to be lifted from the metallic substrate and transferred to the real substrate. To improve the adhesion, a binding material is applied which would affect the performance of the graphene. The production cost is high and the transfer process is difficult to ensure good bonding. Also, it is difficult to apply graphene to a large area over a substrate rather than Cu and Ni. We have developed a special organic ink material that can be printed on any substrate such as polymer and glass. Then we used a laser at UV wavelength to transfer the material to graphene without affecting the substrate. Another process we have developed is the production of large-area graphene using olive oil on glass. We applied laser direct writing of graphene for the production of the electrode in Li–S battery that showed two times the higher electrical capacity with rapid charging (3C) than that of current commercial Li-ion batteries with graphite electrode and retained almost 100% charge capacity even after 500 cycles. Another application of laser direct writing of graphene that we have shown is to produce strain sensors directly on textile material. Both have been published. The difficulties that we came across were the understanding of chemical processes and atomic level reactions involved. It is multiple disciplinary research requiring not only engineering, physics, material science but also chemistry.

**Figure:**
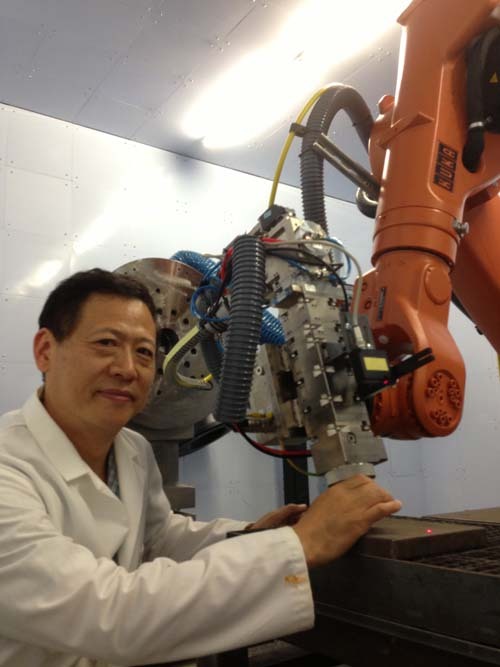
Professor Lin Li in the laboratory

**4. From your research paper published in** ***Light: Science & Applications*** **(light) in 2013**^1^**, a method for direct white-light optical observation of 75-nm adenoviruses was demonstrated for the first time by coupling a standard optical microscope with a BaTiO**_**3**_ **microsphere in water without fluorescent labeling. This work opens new opportunities for the study of virus/cell/bacteria/drug interactions to better understand the causes of various diseases. Against the backdrop of the current COVID-19 Pandemic, this technology seems to carry extra significance. Has this technology been put into the application?**

Lin Li: Our team has commercialized this technology in a spinout company: LIG Nanowise Ltd, in Manchester, UK, to have developed the world’s first commercial optical nanoscope (Nanoro-M model) with super-resolution microsphere amplifying lens (SMAL) that has been sold worldwide including USA, Japan, China, Russia, and South Korea. We have realized super-resolution and high-resolution imaging with large areas at the same time. This is often difficult to achieve. Often a higher resolution microscopic imaging is associated with smaller imaging areas. Examples of real commercial applications of our optical nanoscope include semiconductor inspection by Samsung and credit card examination by Beijing Credit Card Testing Centre. The technology overcomes the theoretical optical diffraction limit of 200 nm in visible light and has realized a 50 nm direct optical resolution with white light and 25 nm with a single wavelength. We are currently collaborating with medical scientists and material scientists to study the interaction of coronaviruses with their detectors and personal protection materials at The University of Manchester. Recently, Nikon, the world’s top microscope company, has announced that they will collaborate with LIG Nanowise Ltd to integrate our SMAL lens technology with Nikon optical microscopes to enable super-resolution imaging for the mainstream market.

**5. As an editor of the Light journal, you have witnessed the growth and development of Light from the beginning. What are your expectations and suggestions for Light, as well as its new sister journals eLight and Light: Advanced Manufacturing?**

Lin Li: “Light” has made history in establishing its high reputation in the field worldwide. The new Light series journals will certainly attract more scientists and engineers to publish. I sincerely wish the new Light series journals success in the coming years.

**Figure:**
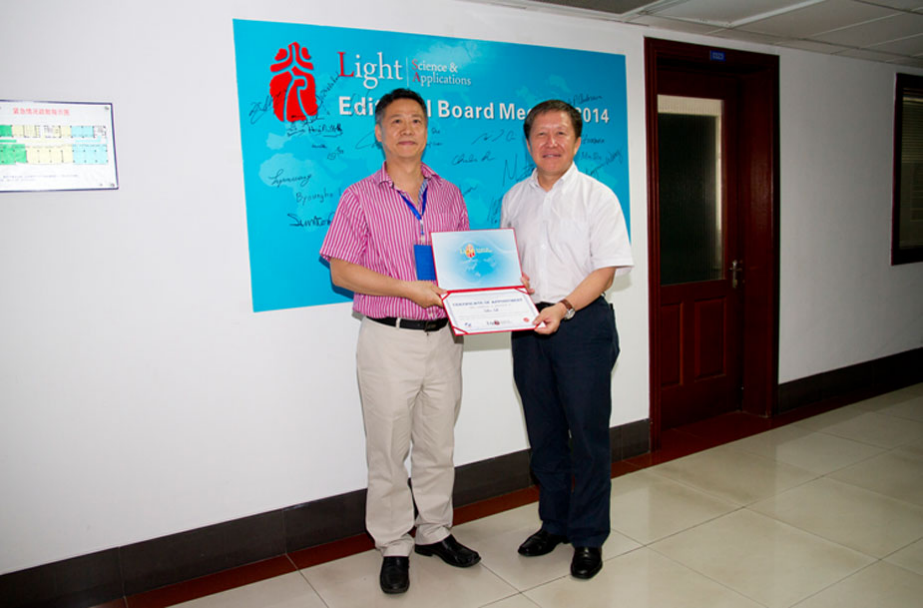
Prof. Cao Jianlin, Editor-in-Chief of the journal, awarded the letter of appointment as a Light editor to Professor Lin Li in 2014.

**6. You are a fellow of the Royal Academy of Engineering, and also have served as president of the Laser Institute of America (LIA), Association of Industrial Laser Users (AILU), and the International Academy of Photonics and Laser Engineering (IAPLE). You are also a fellow of the International Academy of Production Engineering (CIRP) and many other important organizations and have chaired many conferences over the past decades. Could you please share with us some valuable experiences from your work in these associations, and what do board members of these associations value most?**

Lin Li: These international associations and societies are serving the international professional communities in the lasers, optics, and advanced manufacturing fields. My participation in these professional societies enabled me to serve the communities to promote laser science and engineering and knowledge exchange. I have learned a lot from colleagues and friends. The key elements in serving in these professional societies are enthusiasm, sacrifice, dedication, responsibility, and fairness.

**7. You have supervised more than 60 Ph.D. students who are now working in different countries and different fields. You always encourage students to innovate and stand at the forefront of scientific development. What qualities do you think young researchers should possess? Do you have any advice for them?**

Lin Li: Training Ph.D. students is a transformation process. We do not expect them to know everything and be able to solve all the problems at the start. What we expect is that the students should be willing to work hard, willing to face failure and not to be deterred by failure, and have hopes and a desire to succeed. Research is fun and can also be boring. There is a will there is a way. Some of the important inventions may be the result of failed work of the planned research goals. Failure is the mother of success.

**8. Before you joined The University of Manchester, you obtained your Ph.D. degree from the Imperial College London on the topic of laser processing, and you worked with your supervisor Professor William Steen at The University of Liverpool for six years, assisting him in building a new laser processing laboratory. What made you interested in the research of laser processing when you started your Ph.D. program? How did those six years at The University of Liverpool affect your academic career?**

Lin Li: My first degree at the Dalian University of Technology was in automatic control. My initial Ph.D. research topic at Imperial College was on output feedback control theory in the Department of Electrical Engineering. Although the pure theoretical study was useful, and I completed the theoretical work within a few months, I thought more practical, hands-on experience would be more beneficial for a Chinese researcher. One day I met a friend, Mr. Zhenda Chen, who was a Ph.D. student on laser alloying in Dr. Bill Steen’s laser group. I was fascinated by the magic power of lasers, little known at the time for material processing. I applied to the university to change my Ph.D. topic and the supervisor to start my Ph.D. on the adaptive control of the laser cladding process. My Ph.D. thesis title was “Design and Construction of an Intelligent Laser Cladding Control System”, with supervisors from the Department of Materials and Department of Mechanical Engineering. In my Ph.D. study, I applied artificial neural networks and in-process sensors for the control of laser cladding processes between 1985 and 1989. This topic is still a challenge today after 35 years in laser material processing. My postdoctoral research at Liverpool played a very important part in my career. I worked in a number of different projects including in-process monitoring of high-speed (1 m/s) laser welding process for packaging collaboration with a company and laser-based nuclear decommissioning, collaborating with industry. These projects opened up my minds to wider laser processing fields which allowed me to establish my research group in laser processing later on at Manchester.

**9. You are often invited as a plenary speaker to various academic seminars and conferences held in China. Could you please talk about the similarities and differences in laser processing research in and outside of China in recent years?**

Lin Li: China’s laser processing research is world-class and we see a large number of publications in this field coming from China. Between the 1980s and 1990s China had been working mainly in laser cladding, surface treatments, and laser welding and had the highest number of publications in laser cladding. Over the last 20 years, with the availability of more advanced laser facilities in university laboratories and increased international communications, China has quickly progressed into the fields of ultrafast laser micro/nano processing and additive manufacturing. Industrial applications of laser cutting, laser welding, laser marking, and additive manufacturing have boosted the Chinese laser processing industry and research. Internationally, research is mainly in welding, cutting of CFRP composites, welding of aluminum for battery and car production, additive manufacturing of metallic materials, and micro/nano processing of brittle materials such as glass, ceramic and silicon, and synthesis of nano-materials. In many laser processing fields, China is internationally leading. For example, additive manufacturing of very large metallic components by blown powder process, applications of two-photon polymerization for micro/nano device manufacture, laser shock peening, ultra-fast laser surface micro/nano texturing and applications, understanding of electron dynamics in ultra-fasting laser processing, electric current stir assisted laser welding, magnetic field-assisted laser surface cladding and alloying, ring beam laser cladding and multiple laser beam powder bed fusion large metallic component additive manufacturing.

**10. COVID-19 has caused a huge impact on our lives. Many scientific research projects have been interrupted or delayed. Students have had to take online courses. What are your feelings about these changes brought by the pandemic? Could you give some suggestions or advice to help researchers go through this extraordinary period?**

Lin Li: The UK has suffered badly during the Covid-19 pandemic. We have had several national and regional lockdowns. With the wide administration of vaccines, I hope the world will overcome this pandemic soon. We have to change our teaching to online delivery and discussions. We would have to quickly learn and use online teaching tools and develop new online teaching materials and student assessments. We have adopted quickly by working harder. In research and communications, the scale of laboratory work is reduced due to extra safety and social distancing requirements. We have to learn the new operating measures, rules, and limitations. Online meetings are getting more frequent. It is also a lot easier to attend and arrange online meetings. There is no traveling and we have saved a lot of time and it is good for the environment too. This may be integrated into the future mode of communication that more full or partial online meetings and conferences may become normal for international conferences and meetings.

**11. In addition to being a celebrated scientist, a good teacher, and a hardworking team leader, I learned that you are also an outstanding musician who can play a multitude of musical instruments. Your regular BBQ gatherings with your students are also famed. What tips would you give young scientists on how to balance work and life?**

Lin Li: After all, we are human beings and not machines. We have feelings and have lives outside work. I play Chinese flute and erhu as a hobby. I was in the school music band during my middle and high school studies. I participated in the London Chinese Orchestra during my Ph.D. study in the 1980s and performed regularly in the public and now in the Oriental Breeze Chinese music band in Manchester, performing regularly in public. My advice to young people is that enjoy what life has offered and success in work is not always proportional to the time devoted. Balance in work and life will make both work and life more enjoyable.

**Figure:**
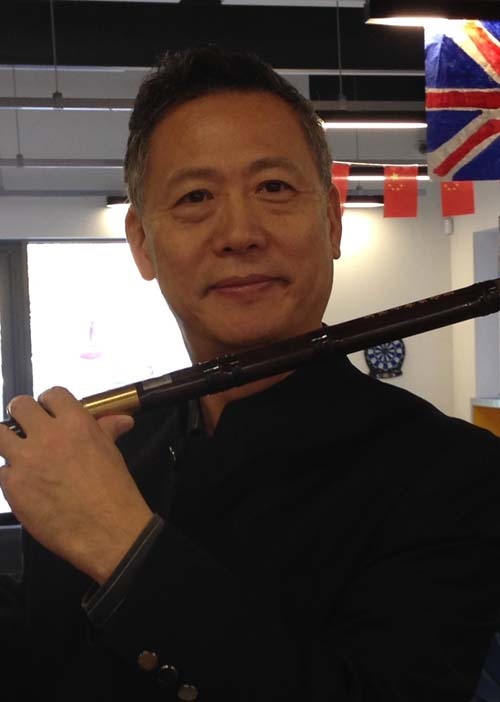
Professor Lin Li plays the Chinese flute

**Figure:**
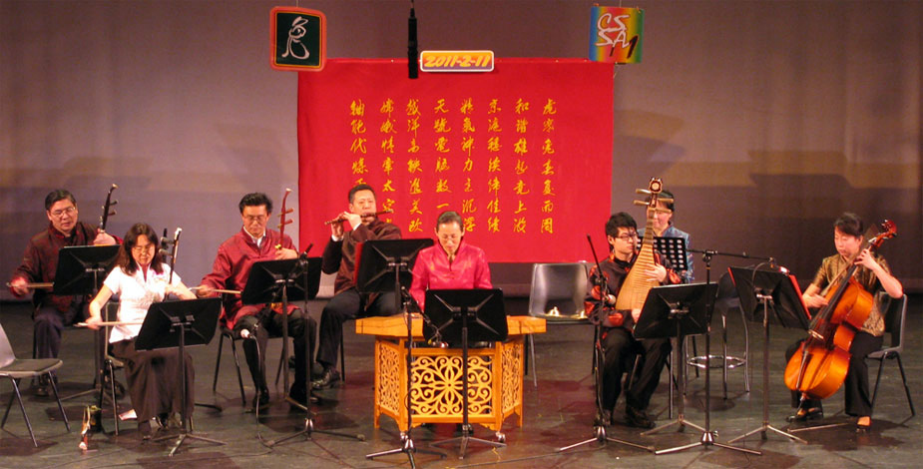
In February 2011, Professor Lin Li (fourth from left) performed in the Oriental Breeze Chinese music band in Manchester.

**12. Your daughter apparently is an excellent student at Imperial College London majoring in medicine. As an alumnus of your daughter, did you give her any advice when she chose university? How do you help her with study and life?**

Lin Li: We did not influence her choice of universities and discipline of study for university. We respected her own choice. She was born, grown, and educated entirely in the UK. Both the schools and the parents encouraged her to independent thinking and decision making. She was determined to do medicine to save lives and care for others, although the education will take a much longer time (6 years instead of 3–4 years for other subjects) and cost more money (She will have to borrow more from the UK government and pay back later). Fortunately, she did well in her A-level examinations with A*s in all the subjects. We are glad that she got what she wanted. She is now very happy and busy studying medicine at Imperial College London where I graduated. In life, we also respect her own choices and give our moral support for her decisions. We still, however, provide some financial support to her study, which is necessary, as the government loan only covers tuition fees and accommodation costs. She communicates with her mum every day by phone.

**Figure:**
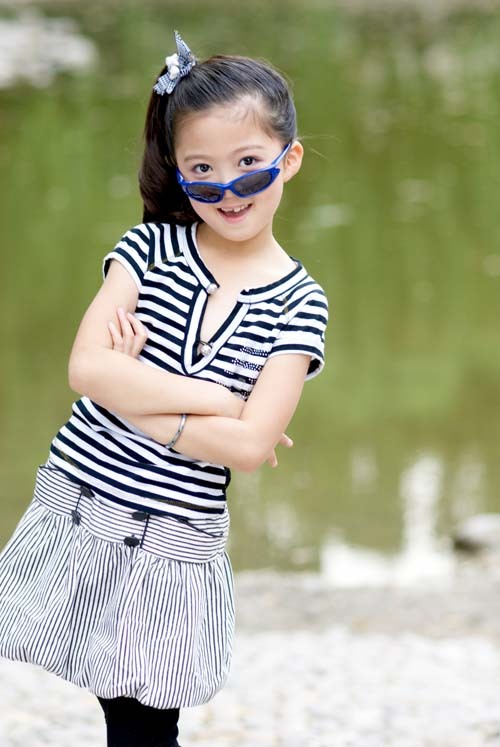
Prof Lin Li’s daughter in her childhood.

**Light special correspondent**

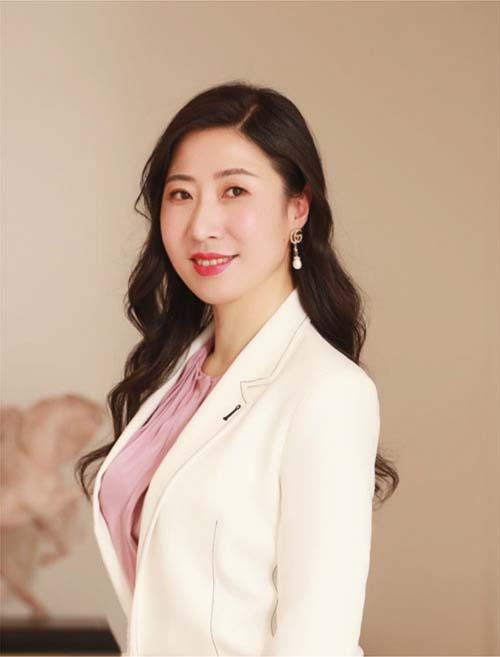



*Hui Wang is the Deputy Director of the Office of International Cooperation in the Changchun Institute of Optics, Fine Mechanics and Physics (CIOMP), Chinese Academy of Sciences (CAS). She currently works on international communication and cooperation for the CIOMP and was a founding member for the Nature Publishing Group and CIOMP joint journal Light: Science and Applications. She is the founder of “Rose in Science” and has published several articles in Acta Editologica, International Talent, Light: Science and Applications, etc., and was invited to take an interview by SPIE Women in Optics, which was published in 2015.*

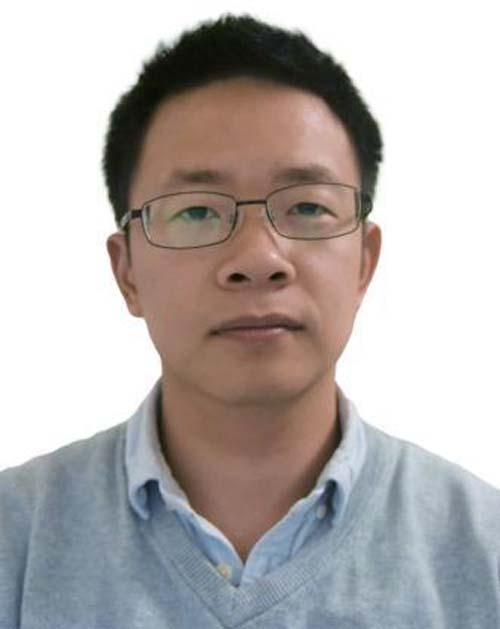



*Heng Gu is a qualified professor in the School of Mechanical Engineering, Jiangsu University. He graduated from The University of Manchester, UK, in 2018, under the supervision of Professor Lin Li. Heng held a postdoctoral position in the ASTUTE project team funded by the European Regional Development Fund at Cardiff University, UK, from 2018 to 2020. He worked with local Welsh companies (including Airbus, Continental, etc.) on a range of projects. His research interests focus on laser material processing such as laser metal deposition, laser welding, and multi-material additive manufacturing. He has published more than 10 papers in journals including the International Journal of Heat and Mass Transfer, Additive Manufacturing, Optics and Laser Technology, and has served as a reviewer for several international journals.*
